# OCGene: a database of experimentally verified ovarian cancer-related genes with precomputed regulation information

**DOI:** 10.1038/cddis.2015.380

**Published:** 2015-12-31

**Authors:** Y Liu, J Xia, J Sun, M Zhao

**Affiliations:** 1 Faculty of Science, Health, Education and Engineering, University of the Sunshine Coast, Maroochydore DC, Sippy Downs, Queensland, Australia; 2 Institute of Health Sciences, Anhui University, Hefei, China; 3 School of Biomedical Informatics, The University of Texas Health Science Center at Houston, Houston, TX, USA

Dear Editor,

Ovarian cancer (OC) is a heterogeneous cancer primarily beginning in an ovary. It often results in tumor cells that can metastasis to invade to other organs of human body. In 2012, it was reported 239 000 new cases and 152 000 deaths worldwide.^1, 2^ As the eighth-most common cause of death, OC is regarded as a ‘silent killer' due to absence of symptoms in early stages.^1, 2^ Integrating literature evidence is critical for cross-validation of cancer mechanisms. During the past decade, thousands of small-scale studies have been published regarding the OC-related genes and how they function in various cellular events. Unfortunately, there is no such an integrated resource for OC genes with literature evidence available. Here, we developed OCGene with substantial literature evidences and regulatory data at http://ocgene.bioinfo-minzhao.org/.

The gene collection on the OC were mainly based on 12 data sources, including OMIM, GAD, one online database focusing on OC genes,^3^ one expert review,^4^ gene manually curation from GeneRif database, genome-wide association studies from GWASCatalog and six candidate gene lists produced by various large-scale genomic platforms on OC from the TCGA^5^ (detailed in the Supplementary Methods). In total, 2067 genes associated with 2819 PubMed abstracts were compiled. The comprehensive functional annotations were associated with all the genes, including gene expression profiles, homologous genes in other model species, protein–protein interaction, somatic mutations and methylations.

As shown in Figure 1a, a typical OCGene gene entry contains eight categories information by clicking the label ‘General information,' ‘Literature,' ‘Expression,' ‘lncRNA,' ‘Regulation,' ‘Mutation,' ‘Homolog' and ‘Interaction' on the top. Highlighted summaries of curated literature evidence are provided in the ‘Literature' page. For the precomputed coexpression pattern between OC-related genes and lncRNAs, user can obtain the correlation coefficients and the corrected statistical *P*-values. In addition, the expression plots of the corresponding lncRNAs were also integrated from MiTranscriptome database^6^ (detailed in the Supplementary Methods). The ‘Homolog' page is used to map those human genes with evidence to other model species, including mouse, rat, zebrafish and fruit fly. Moreover, the ‘Regulation' page is used to classify regulatory information, including interactions with transcription factors, abundance of post-translational modification information and methylation information.

On the basis of the gene ranking results of ToppGene^7^ (detailed in the Supplementary Methods), the top 100-ranked genes tend to have multiple evidences from different data sources (Figure 1b). These highly overlapping relationship of top-ranked genes support the accuracy of our data. In addition, using reliable pathway-based gene interaction data, we constructed a more comprehensive cellular map for OC based on top 100-ranked genes (Figure 1c). Of the 79 nodes, 74 of them are from our OCGene. The remaining five are the novel genes that may potentially bridge the OC-related gene to fully implement their cellular function. In conclusion, the majority of curated top-ranked OC-related genes are linked to each other in a highly modular structure, which may accelerate the signaling processing for oncogenesis.

In conclusion, we developed a user-friendly literature-based online database for in-depth analysis of OC genes with comprehensive annotation. The features of our database include: (i) manually curated experimentally verified genes from thousands of literatures; (ii) comprehensively integrated from nine reliable data sources; and (iii) precomputed regulatory pattern involved transcription factors, microRNA and long non-coding RNAs. These comprehensive annotations and precomputed regulatory information highlighted the importance of OCGene to elucidating unexplored field of OC development. All the information with functional features are available in a plain text format that is free to download.

## Figures and Tables

**Figure 1:**
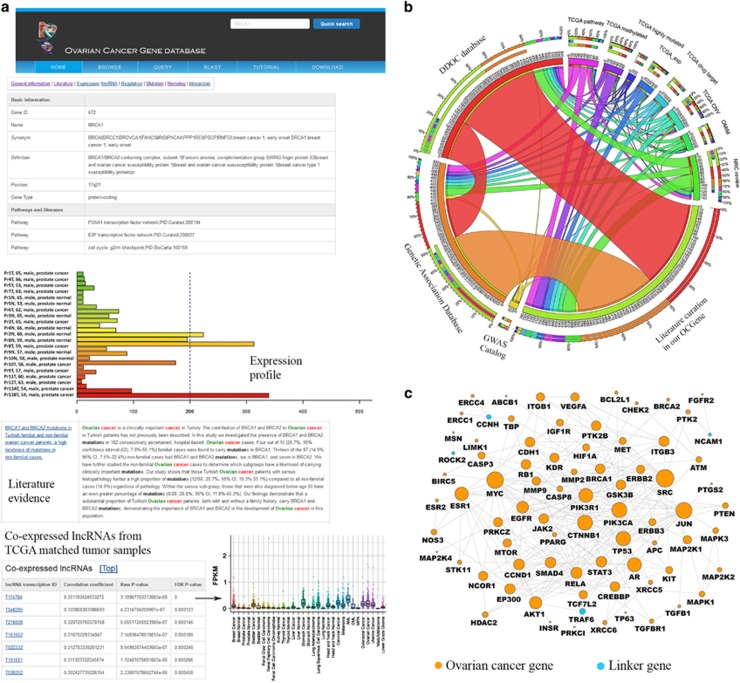
(**a**) Web interface of OCGene, including the basic information, gene expression, curated literatures and precomputed lncRNA coexpression results, using TCGA ovarian cancer tumor samples. (**b**) The shared OC-related genes across multiple data source. The length of circularly arranged segments is proportional to the total genes in each data source. The ribbons connecting different segments represent the number of shared genes between data sources. The three outer rings are stacked bar plots that represent the relative contribution of other data sources to their totals. (**c**) Reconstructed cellular map using the top-100-ranked OC-related genes based on protein–protein interaction from pathway database. The 74 genes in orange are genes from the top-100-ranked OC-related genes. The remaining five genes in blue are linker genes that bridge the 74 genes
