# Supplementary material for: OCGene: a database of experimentally verified ovarian cancer-related genes with precomputed regulation information
Source: Cell Death Dis. 2015 Dec 31;6(12):e2036–. doi: 10.1038/cddis.2015.380 (PMC4720911; doi:10.1038/cddis.2015.380)
Supplement: Supplementary Information [file cddis2015380x1.doc]

**Supplementary methods**

**OCGene: a database of experimentally verified ovarian cancer-related genes with pre-computed regulation information**

Yining Liu^1^, Junfeng Xia^2^, Jingchun Sun^3^, Min Zhao^1§^

^1^Faculty of Science, Health, Education and Engineering, University of the Sunshine Coast, Maroochydore DC, Queensland, 4558, Australia.

^2^Institute of Health Sciences, Anhui University, Hefei 230601, China.

^3^School of Biomedical Informatics, The University of Texas Health Science Center at Houston, Houston, TX 77030, USA.

^§^Corresponding author

Email addresses: [mzhao@usc.edu.au](mailto:mzhao@usc.edu.au)

**Methods**

**Gene collection**

The gene collection on the ovarian cancer (OC) were mainly based on nine data sources, including Online Mendelian Inheritance in Man (OMIM) ^1^, genetic association database (GAD) ^2^, Dragon Database for Exploration of Ovarian Cancer Genes (DDOC) database focusing on ovarian cancer genes ^3^, one expert review from Nature review cancer ^4^, gene manually curation from GeneRif ^5^, genome-wide association studies from GWASCatalog ^6^ and six candidate gene lists produced by various large-scale genomic platforms on OC from the TCGA ^7^. In detail, we collected 19 genes from OMIM by using the keyword searching of ovarian cancer: *BRCA1, ERBB2, BRCA2, PIK3CA, MLH1, AKT1, CTNNB1, CDH1, DIRAS3, RAD51C, OPCML, RBBP8, PARK2, ST8, AGO2, DICER1, OVCAS1, RRAS2,* and *SEPT9*. From GAD database, a total of 558 genes are collected based on the well classified ovarian cancer phenotype. From DDOC database (<http://apps.sanbi.ac.za/ddoc/>), we collected 379 genes with experimentally verified information. From the expert review published in Nature review cancer, we included 51 additional genes. From GWASCatalog database, we harvested a total of 62 genes associated with ovarian cancer.

For the TCGA nature paper on ovarian cancer, all the data are downloaded at the following URL: <http://tcga-data.nci.nih.gov/docs/publications/ov_2011/>.

In total, six gene lists from the paper and its supplement are compiled as below.

| **Dataset name** | **# of genes** | **Description** |
| --- | --- | --- |
| 7_highly_mutated_genes | 7 | 7 significant mutated genes (P-value<10-4) were collected |
| 125_from_26_CNV_regions | 125 | 26 regions of focal amplification that encoded eight or fewer genes. |
| 168_methylated_genes | 168 | Epigenetically silenced genes. |
| 193_prognostic_signature | 193 | 193 signature genes to predict overall survival that was defined using the integrated expression data. The 108 of the 193 genes were correlated with poor survival and the rest 85 were correlated with good survival. |
| 22_drug_target_S5.3 | 22 | Curated genes with therapeutic compounds from Table S5.2. |
| 34_pathway_analysis | 34 | Genes in altered pathways in the Figure 3. Although the supple 9 contains more genes, its content is really confused. Thus, I only collected genes in the Figure 3 in its main manuscript. |

The five gene set from TCGA have overlapping to each other. By removing the redundant, we collected 520 genes in total.

To further collect the newly published genes from literature, we used the GeneRif database (<http://www.ncbi.nlm.nih.gov/gene/about-generif>). We downloaded the data on Apr 5, 2015, we collected the references by using the Perl regular expression to match those sentences with both ovary and cancer keywords: [(ovarian OR ovary) AND (cancer OR tumor OR carcinoma)]. Based on the selected reference, we did the manually curation by collecting gene names from the descriptions of the text and mapping the gene names to Entrez gene IDs. To obtain more accurate literature evidence, we collected the species information and the gene alias and manually mapped to official HUGO gene symbol. For example, in the sentence " *COX-2 overexpression may be significantly related to the oncogenesis and development of Ovarian serous carcinoma, which may be an early diagnostic parameter and, hence, an attractive target for chemopreventive strategy in the treatment of Ovarian serous carcinoma* ” ^8^ the gene name *COX-2* was the common alias of *PTGS2* in the current NCBI Entrez gene database. For the genes implicated in other model species, we mapped all the curated genes to their corresponding human homologous groups using NCBI HomoloGene database as we implemented as previous analysis ^9, 10, 11^. Finally, 1260 genes from 2571 positive Pubmed abstract were collected.

By integrated all the genes from the six data resources, we integrated a total of 2068 OV-related genes associated with 2825 PubMed abstracts.

**Bioinformatics annotation**

The basic gene information and sequences are included and crosslinked to the NCBI Entrez gene and Homologene database (Downloaded on Apr 8, 2015) ^12^. The mRNA expression profiling data from both normal and tumor tissues are imported from BioGPS ^13^. The expression values of BioGPS database are based on Affymetrix chips relate to fluorescence intensity. Since there are multiple probes for each transcript on the microarray, these intensity values are summarized using the data processing algorithm GCRMA ^14^. To provide an overview of the most prevalent cancer mutations, we annotated all the TSGs using the mutational data from COSMIC (V72) ^15^. To obtain comprehensive pathway-related information, we annotated the OC-related genes using BioCyc ^16^ and KEGG Pathway ^17^. The other useful regulation information include post-translational modification ^18^, methylation ^19^, and protein-protein interaction from Pathway Commons (V5) ^20^. All the included functional or genomic features are seamlessly integrated to produce a downloadable output in a plain text format. More annotation data will be added to OCGene when it becomes available.

**Web interface**

All data and annotation information in OCGene are stored in a MySQL-based database on a Linux server. OCGene has two main views of search results: text-based query and sequence-based BLAST search. The text-based query can help users to retrieve a list of genes with annotations of their interest. The sequence-based BLAST is used to annotate unknown genes. Furthermore, users can browse the data in a variety of ways, including co-expressed lncRNAs, annotated tumor suppressor genes from TSGene database ^10^, highlighted KEGG pathway, and genomic positions. By applying our previously implemented computational framework on microRNA and transcription factor co-regulation motif ^21^, we provide the pre-computed regulatory information for advanced systems biology analysis.

**Gene Ranking**

To prioritize the relative importance for all the curated genes, we compiled a training gene list that included 23 genes with at least 20 literature evidence to train the ranking model of ToppGene ^22^. The top 100 ranked genes often have multiple evidences from different data sources. These highly overlapping relationship of top ranked genes support the accuracy of our data.

**Co-expressed long non-coding RNAs from TCGA matched cancer samples**

To provide a list of co-expressed long non-coding RNAs (lncRNAs) of OC-related genes, we collected all lncRNAs expression data from one of the recent developed database Mitranscriptome ^23^. The Mitranscriptome contains the assembled lncRNAs based on the RNAseq data from thousands of TCGA tumor samples. We calculated the expression correlation among OC-related genes and all 17,250 lncRNAs from Mitranscriptome using Spearman’s correlation using the matched The Cancer Genome Atlas (TCGA) ovarian cancer samples ^7, 24^. The correlation coefficients and corresponding significant *P* values were calculated using R (version 2.14.0), and a false discovery rate (FDR) was applied to correct for multiple testing. For all OC-related gene and lncRNA pairs, we required expression correlation scores greater than 0.3 and FDR adjusted *P* values less than 0.01.

**Database update plan**

To update of relevant literatures in future, we constructed an automatic literature searching term using My NCBI tool, which will retrieve PubMed references every two weeks based on the keyword matching. The Entrez reference similarity will be used to cluster the newly available reference to those curated references in our OCGene. Additionally, we also implemented an automatic system to annotate the ovarian cancer-related genes by integrating functional information from public data sources. Once the data content update, the web interface will be updated accordingly annually.

**References**

1. Amberger J, Bocchini CA, Scott AF, Hamosh A. McKusick's Online Mendelian Inheritance in Man (OMIM). *Nucleic Acids Res* 2009, **37**(Database issue)**:** D793-D796.

2. Becker KG, Barnes KC, Bright TJ, Wang SA. The genetic association database. *Nat Genet* 2004, **36**(5)**:** 431-432.

3. Kaur M, Radovanovic A, Essack M, Schaefer U, Maqungo M, Kibler T*, et al.* Database for exploration of functional context of genes implicated in ovarian cancer. *Nucleic Acids Res* 2009, **37**(Database issue)**:** D820-823.

4. Bast RC, Jr., Hennessy B, Mills GB. The biology of ovarian cancer: new opportunities for translation. *Nat Rev Cancer* 2009, **9**(6)**:** 415-428.

5. Aronson AR, Mork JG, Gay CW, Humphrey SM, Rogers WJ. The NLM Indexing Initiative's Medical Text Indexer. *Stud Health Technol Inform* 2004, **107**(Pt 1)**:** 268-272.

6. Welter D, MacArthur J, Morales J, Burdett T, Hall P, Junkins H*, et al.* The NHGRI GWAS Catalog, a curated resource of SNP-trait associations. *Nucleic Acids Res* 2014, **42**(Database issue)**:** D1001-1006.

7. The Cancer Genome Atlas Network. Integrated genomic analyses of ovarian carcinoma. *Nature* 2011, **474**(7353)**:** 609-615.

8. Tang LX, Wang M, Ma JW. [Relation between COX-2 protein expression and biologic behavior of ovarian carcinoma]. *Zhonghua Zhong Liu Za Zhi* 2003, **25**(3)**:** 261-263.

9. Brown GR, Hem V, Katz KS, Ovetsky M, Wallin C, Ermolaeva O*, et al.* Gene: a gene-centered information resource at NCBI. *Nucleic Acids Res* 2015, **43**(Database issue)**:** D36-42.

10. Zhao M, Sun J, Zhao Z. TSGene: a web resource for tumor suppressor genes. *Nucleic Acids Res* 2013, **41**(Database issue)**:** D970-976.

11. Kong L, Cheng L, Fan LY, Zhao M, Qu H. IQdb: an intelligence quotient score-associated gene resource for human intelligence. *Database (Oxford)* 2013, **2013:** bat063.

12. Sayers EW, Barrett T, Benson DA, Bolton E, Bryant SH, Canese K*, et al.* Database resources of the National Center for Biotechnology Information. *Nucleic Acids Res* 2011, **39**(Database issue)**:** D38-51.

13. Su AI, Wiltshire T, Batalov S, Lapp H, Ching KA, Block D*, et al.* A gene atlas of the mouse and human protein-encoding transcriptomes. *Proc Natl Acad Sci U S A* 2004, **101**(16)**:** 6062-6067.

14. Gharaibeh RZ, Fodor AA, Gibas CJ. Background correction using dinucleotide affinities improves the performance of GCRMA. *BMC Bioinformatics* 2008, **9:** 452.

15. Forbes SA, Beare D, Gunasekaran P, Leung K, Bindal N, Boutselakis H*, et al.* COSMIC: exploring the world's knowledge of somatic mutations in human cancer. *Nucleic Acids Res* 2015, **43**(Database issue)**:** D805-811.

16. Karp PD, Ouzounis CA, Moore-Kochlacs C, Goldovsky L, Kaipa P, Ahren D*, et al.* Expansion of the BioCyc collection of pathway/genome databases to 160 genomes. *Nucleic Acids Res* 2005, **33**(19)**:** 6083-6089.

17. Kanehisa M, Araki M, Goto S, Hattori M, Hirakawa M, Itoh M*, et al.* KEGG for linking genomes to life and the environment. *Nucleic Acids Res* 2008, **36**(Database issue)**:** D480-484.

18. Lee TY, Huang HD, Hung JH, Huang HY, Yang YS, Wang TH. dbPTM: an information repository of protein post-translational modification. *Nucleic Acids Res* 2006, **34**(Database issue)**:** D622-627.

19. Lv J, Liu H, Su J, Wu X, Li B, Xiao X*, et al.* DiseaseMeth: a human disease methylation database. *Nucleic Acids Res* 2012, **40**(Database issue)**:** D1030-1035.

20. Cerami EG, Gross BE, Demir E, Rodchenkov I, Babur O, Anwar N*, et al.* Pathway Commons, a web resource for biological pathway data. *Nucleic Acids Res* 2011, **39**(Database issue)**:** D685-690.

21. Zhao M, Sun J, Zhao Z. Synergetic regulatory networks mediated by oncogene-driven microRNAs and transcription factors in serous ovarian cancer. *Mol Biosyst* 2013, **9**(12)**:** 3187-3198.

22. Chen J, Bardes EE, Aronow BJ, Jegga AG. ToppGene Suite for gene list enrichment analysis and candidate gene prioritization. *Nucleic Acids Res* 2009, **37**(Web Server issue)**:** W305-311.

23. Iyer MK, Niknafs YS, Malik R, Singhal U, Sahu A, Hosono Y*, et al.* The landscape of long noncoding RNAs in the human transcriptome. *Nat Genet* 2015, **47**(3)**:** 199-208.

24. Cancer Genome Atlas Research Network, Kandoth C, Schultz N, Cherniack AD, Akbani R, Liu Y*, et al.* Integrated genomic characterization of endometrial carcinoma. *Nature* 2013, **497**(7447)**:** 67-73.
